# Paediatric Acute-onset Neuropsychiatric Syndrome (PANS) and intravenous immunoglobulin (IVIG): comprehensive open-label trial in ten children

**DOI:** 10.1186/s12888-022-04181-x

**Published:** 2022-08-06

**Authors:** Parisa Hajjari, Malin Huldt Oldmark, Elisabeth Fernell, Klara Jakobsson, Ingrid Vinsa, Max Thorsson, Mehran Monemi, Lotta Stenlund, Anders Fasth, Catrin Furuhjelm, Jakob Åsberg Johnels, Christopher Gillberg, Mats Johnson

**Affiliations:** 1 Gillberg Neuropsychiatry Centre, Sahlgrenska Academy, University of Gothenburg, Kungsgatan 12A, 411 19 Gothenburg, Sweden; 2 Akutläkarna Specialist Clinic, Gothenburg, Sweden; 3 Department of Pediatrics, Institute of Clinical Sciences, Sahlgrenska Academy, University of Gothenburg, Gothenburg, Sweden; 4 Department of Biomedical and Clinical Sciences, Linköping University, Linköping, Sweden

**Keywords:** Paediatric Acute-onset Neuropsychiatric Syndrome, PANS, Intravenous Immunoglobulin/IVIG

## Abstract

**Background:**

Treatment with intravenous immunoglobulin (IVIG) in children with Paediatric Acute-onset Neuropsychiatric Syndrome (PANS) has for many years been used on clinical indications, but the research evidence for its efficacy is insufficient.

**Methods:**

Open-label prospective in-depth trial including ten children (median age 10.3 years) with PANS, who received IVIG treatment 2 g/kg monthly for three months. Primary outcomes were changes in symptom severity and impairment from baseline to first and second follow-up visits one month after first and one month after third treatment, using three investigator-rated scales: Paediatric Acute Neuropsychiatric Symptom (PANS) scale, Clinical Global Impression – Severity and Improvement (CGI-S and CGI-I) scales. Secondary outcomes reported here were changes in Children’s Yale-Brown Obsessive Compulsive Scale (CY-BOCS) scores, and side effects.

**Results:**

All ten children received three treatments at one-month intervals according to study plan. From baseline to second follow-up marked reductions were seen in mean total PANS scale scores (*p* = .005), and CGI-S scores (*p* = .004). CGI-I ratings showed much to very much global improvement (mean CGI-I 1.8). Nine children had clinical response defined as > 30% reduction in PANS Scale scores. Improvements were also noted for CY-BOCS scores (*p* = .005), and in school attendance. Three children suffered moderate to severe temporary side effects after the first treatment, and the remaining seven had mild to moderate side effects. Side effects were much less severe after second and third treatments.

**Conclusions:**

Considerable and pervasive improvements in symptoms and clinical impairments were seen in these ten children after three monthly IVIG treatments. Moderate to severe transient side effects occurred in three cases.

**Trial registration:**

EudraCT no. 2019–004758-27, Clinicaltrials.gov no. NCT04609761, 05/10/2020.

## Introduction

Paediatric Acute-onset Neuropsychiatric Syndrome (PANS) is characterised by abrupt dramatic onset of obsessive–compulsive disorder (OCD) and/or eating restrictions, combined with at least two other severe neuropsychiatric symptoms. These symptoms may be choreiform movements, separation anxiety, sensory symptoms, severe emotional lability, and hallucinations. Tics and enuresis are also very common symptoms. PANS is a diagnosis of exclusion, meaning that other neurological/medical disorders must be ruled out before a diagnosis can be made [1–5]. Cases with abrupt onset of such symptoms following a streptococcal infection have been described with the term paediatric autoimmune neuropsychiatric disorders associated with streptococcal infections (PANDAS), which is considered to be a subgroup of PANS [1].

The aetiology of PANS is still unknown. A host of possible different underlying mechanisms have been proposed including autoimmunity with neuroinflammation, postinfectious disease and exacerbation/relapse episodes of underlying neurodevelopmental/neurological disorders [1, 3, 6]. Autoimmune/inflammatory disorders in first-degree relatives have been reported in around 50–70% of all PANS cases, and in obsessive–compulsive disorder and Tourette/chronic tic disorders [2, 4, 7].

Recent treatment approaches/recommendations for PANS have included psychiatric and behavioural interventions, e.g. cognitive behaviour therapy [8, 9] despite the lack of systematic studies in the field. Antibiotics, nonsteroid anti-inflammatory drugs (NSAIDs), corticosteroids, plasmapheresis, and intravenous immunoglobulin (IVIG) have also been proposed [10], but here too, more in-depth research and prospective long-term outcome follow-up is needed [11, 12].

Other than longer-term psychiatric/behavioural interventions, IVIG represents one of the most “invasive” forms of therapy proposed for PANS. Performing double-blind intravenous infusion studies using placebo-control in young children affected by a severe neurodevelopmental/psychiatric disorder presents a huge ethical challenge. To our knowledge, only two such studies have been performed, and they have shown conflicting results. One of these trials compared plasma exchange, IVIG and placebo (saline solution) for treatment of exacerbations of neuropsychiatric symptoms in children with infection-triggered OCD and tic disorders and reported that plasma exchange and IVIG were both better than placebo [13]. The other randomized controlled trial (RCT) found no significant difference between the intervention and placebo groups in children with PANDAS [14]. In addition to the ethical problem of treating children with an inactive, intravenously administered placebo, it is also problematic to randomize children with complex neurodevelopmental/neuropsychiatric disorders with different symptom combinations and severities. IVIG infusions also have well-known and tangible transient side effects such as headache and nausea, making it difficult to maintain blinding in an RCT.

There is a lack of IVIG trials measuring long-term functional and quality of life outcomes, as well as safety and tolerability. We are aware of only one study of IVIG in PANS that has included a longitudinal prospective approach and in-depth outcome follow-up [15]. The results of that open-label study – of 21 children aged 4–16 years who received 6 monthly 1 g/kg IVIG treatments—indicated positive behavioural outcomes for a majority of the individuals included. IVIG was well tolerated and mild to moderate side effects were reported. Another open trial including 55 children who received two successive days of 2 g/kg IVIG treatment, reported substantial improvements in OCD symptoms measured by the Children´s Yale-Brown Obsessive–Compulsive Scale (CY-BOCS), lasting for 1 year in 85% of the patients [16], but this trial did not report other symptom or functional outcomes.

We therefore chose to design a prospective longitudinal 18-month in-depth outcome study including symptomatic, functional, quality of life, safety and tolerability outcomes in children and adolescents with narrowly defined PANS treated with IVIG, including assessments by physician, psychologist, study nurse, parents, and school. Here, we present 3-month data from our study after three monthly IVIG treatments.

### Objectives

The primary objective was to evaluate the efficacy of IVIG in improving neuropsychiatric symptoms and impairment.

Secondary objectives comprised evaluation of changes in OCD symptoms, adaptive and cognitive functioning, quality of life, number of school days missed per month, parental care load, safety and tolerability of the treatment. Most of the secondary results will be reported in a separate paper.

## Methods

### Study design and participants

Open-label non-placebo trial with IVIG infusions 2 g/kg given every 4 weeks for 3 months, followed by IVIG doses at 4-week intervals or longer up to a total of 6 IVIG treatments, as needed depending on symptom development, in 10 children and adolescents with post-infectious PANS including the subgroup PANDAS.

After pre-screening assessments of 25 patients consecutively referred from paediatricians/child neurologists/child psychiatrists to the Gillberg Neuropsychiatry Centre (GNC) research project OPHELIA (On PANS: Holistic Epidemiology, Longitudinal Immunotherapies, Additional treatments) in Gothenburg between November 2020 and March 2021, ten children and adolescents (six girls and four boys, age 6 – 16 years) were screened for inclusion in the trial and were all found to be eligible. The other patients either did not meet criteria for PANS or were considered to benefit from other treatments. The project is a collaboration between the GNC, the Specialist Clinic “Akutläkarna” in Gothenburg, and the paediatric clinics in Linköping and Lund. The IVIG treatments were given at the Akutläkarna Clinic or at the child´s local paediatric clinic. The trial was approved by the Swedish Medical Products Agency. 

### Ethics

The trial was approved by the Swedish Ethical Review Authority. Parents/caregivers and patients were informed orally and in writing about the study design, treatment procedures, possible benefits and risks of the treatment, and provided written informed consent or assent, as appropriate.

### Main inclusion criteria

1. Age 4 to 17 years at Baseline.
2. Confirmed pre-existing diagnosis of post-infectious PANS/PANDAS, according to criteria proposed by Swedo et al. [1]
3. No treatment with IVIG during the last 6 months.

### Main exclusion criteria

1. Significant acute or chronic diseases that may interfere with successful completion of the trial or place the subject at undue medical risk.
2. Known serious adverse reaction to immunoglobulin or any severe anaphylactic reaction to blood-derived products. Females of childbearing potential who were pregnant, had a positive pregnancy test at Baseline, were breastfeeding, or unwilling to practice a highly effective method of contraception.
3. Significant proteinuria or nephrotic syndrome, history of acute renal failure, severe renal impairment, aspartate aminotransferase (ASAT) or alanine aminotransferase (ALAT) levels exceeding 2.5 times the upper limit of normal, or hemoglobin < 90 g/L.
4. Medication with immunosuppressants, immunomodulators, long-term systemic corticosteroids (intermittent courses of corticosteroids of not more than 10 days were allowed).
5. Known drug abuse.
6. Participation in another clinical trial within 30 days prior to Baseline.
7. Mentally challenged subjects/families who could not give independent informed consent.

If the patient was on long-term antibiotic prophylaxis, it should be unchanged one month before Baseline and during the trial. Infections occurring during the trial should be treated according to standard clinical practice. If not considered essential for the subject, corticosteroids and NSAIDs should be discontinued at least one month before baseline and during the trial. Any psychopharmacological treatment (e.g. Selective Serotonin Reuptake Inhibitors (SSRI), antipsychotics) should be discontinued at least one month before baseline, or if considered essential for the subject, be kept at a stable and unchanged dose from one month before baseline and during the trial. 

Before study start, if clinically indicated, cerebrospinal fluid analyses, cerebral magnetic resonance imaging and electroencephalogram were performed by referring clinicians to rule out encephalitis and other neurologic conditions.

### Instruments

The PANS scale was developed by Swedo et al. at the National Institute of Mental Health [17]. The scale details the symptoms included in the PANS criteria (see Table 1–2). Each symptom is scored from 0–5 (none to very severe). The OCD part of the scale includes six domains of OCD symptoms, and the score of the most severe domain is multiplied by 5 to give a total OCD score (0–25). The associated neuropsychiatric (NP) symptom part includes seven domains, and the most severe score (0–5) in five of these domains are added to give a NP score (0–25). The sum of the OCD and NP scores yields a total symptom score (0–50). The impairment part of the scale estimates impairment in self-esteem, family life, social acceptance, and school or work functioning, scored from 0–50 (none to extreme difficulties). In our trial, ratings were based on parent and patient interviews.

**Table 1:** Participant demographics

Demographics	Participants *n* = 10
Age at PANS onset. Years, median (range)	7.1 (5.0 − 9.4)
Sex	
Boys	4 (40%)
Girls	6 (60%)
Age at baseline. Years, median (range)	10.3 (6.3 − 16.1)
Duration of illness. Years, mean (range)	4.0 (0.3–9.2)
Preexisting neurodevelopmental disorder	4 (40%)
Preexisting neurodevelopmental symptoms	2 (20%)
No neurodevelopmental disorder/symptoms	4 (40%)
Autoimmune diseases in first- or second-degree relatives	7 (70%)
Streptococcal infection preceding PANS onset	
Verified/colonized	2 (20%)
Suspected	3 (30%)
CGI-S at baseline (Mean, SD)	5.8 (0.42)
PANS Scale (Mean, SD)	
Symptom	41.4 (5.30)
Impairment	39.8 (3.39)
Total	81.2 (7.83)
Concomitant medication:	
SSRI	6 (60%)
Antipsychotics	3 (30%)
ADHD medication	3 (30%)
Antibiotics	3 (30%)
NSAIDS	2 (20%)
Sleeping medication	5 (50%)
Antihistamines	1 (10%)

CGI-S (Clinical Global Impression scale-Severity). PANS (Paediatric Acute Neuropsychiatric Symptom) scale

SSRI (Selective Serotonin Reuptake Inhibitors). NSAIDs (Nonsteroidal anti-inflammatory drugs)

**Table 2:** PANS symptom profiles at Baseline

PANS symptoms	Presence of symptoms at baseline in percent (*n* = 10)	Mean value PANS score
**Major PANS criteria**		
**Obsessive- compulsive symptoms**	10 (100%)	3.8
**Eating restriction**	8 (80%)	2.4
**Minor PANS criteria**		
**1. Anxiety symptoms**		
Separation anxiety	9 (90%)	2.9
General anxiety	10 (100%)	3.6
Irrational fears/phobias	8 (80%)	3.0
Panic episodes	9 (90%)	2.5
**2. Emotional lability, depression**		
Emotional lability, mood swings	10 (100%)	3.8
Depression with/without suicidal/self-injurious thoughts	8 (80%)	2.2
**3. Increased irritability or aggressive behavior**	10 (100%)	3.9
**4. Behavioral regression**		
Behavioral regression (behavior atypical for actual age)	8 (80%)	2.4
Change in personality	10 (100%)	3.3
**5. School performance, concentration/learning**		
Difficulties in attention, concentration or learning	9 (90%)	3.0
Loss of academic skills (math, reading, writing)	8 (80%)	3.1
Confusion	0%	0
**6A. Sensory symptoms**	10 (100%)	3.6
**6B. Hallucinations**	2 (20%)	0.7
**6C. Motor symptoms**		
Dysgraphia	6 (60%)	1.9
Motoric hyperactivity	9 (90%)	3.0
Piano playing movements	2 (20%)	0.2
Simple motor/vocal tics	7 (70%)	2.0
Complex motor/vocal tics	4 (40%)	0.9
**7A. Urinary symptoms**	4 (40%)	1.0
**7B. Sleep disturbance, fatigue**		
Sleep problems	7 (70%)	1.8
Extreme tiredness or fatigue	8 (80%)	2.7
**7C. Dilated pupils**	7 (70%)	1.7
**Body pain**	7 (70%)	

The PANS symptom severity scores are rated between 0 (no symptoms) and 5 (extreme symptoms)

The Clinical Global Impression (CGI) scales [18] have been used in numerous clinical trials. Here, rating was based on interviews with parents and child considering symptoms and functional impairment according to all available information. CGI-Improvement (CGI-I) rates the global development of the patient’s condition compared to baseline, scored from 1—very much improved, 2—much improved, 3—minimally improved, 4—no change, 5—minimally worse, 6—much worse, to 7—very much worse. CGI-Severity (CGI-S) rates global symptom severity on a scale from 1 to 7; 1—not at all ill, 2—borderline, 3—mildly ill, 4—moderately ill, 5—markedly ill, 6—severely ill, and 7—extremely ill. Clinical response is defined by CGI-I or CGI-S ratings of 1–2.

The Children´s Yale-Brown Obsessive–Compulsive Scale (CY-BOCS, [19]) is a widely used instrument designed to assess obsessive–compulsive disorder symptoms in children and adolescents aged 6—17 years. CY-BOCS is a semi-structured clinician-administered interview to assess obsession and compulsion severity over the previous week. The scale consists of 10-items rated on a 5-point Likert scale ranging from 0 (no symptoms) to 4 (severe symptoms) and yields a total severity score between 0—40. The scale has been used in multiple treatment trials. Positive treatment response has been defined as a 25% reduction in total score, and a total score of < 15 is considered as diagnostic remission [20].

### Primary outcomes

Changes in symptom severity and impairment on the investigator-rated PANS scale. Clinical response was defined as > 30% reduction in symptoms and impairment.

Changes in global symptoms and functioning measured by CGI-S, and in global improvement measured by CGI-I. Clinical response was defined as a score of 1–2 on CGI-S and CGI-I, respectively.

### Secondary outcomes published here

OCD symptoms measured with the CY-BOCS scale.

Days absent from school per month during the study period.

Adverse Events (AEs), Serious AEs (SAEs), and discontinuations due to AEs and SAEs.

### Secondary outcomes not published here

The following secondary outcome measures will be presented in forthcoming publications:

- Assessments of adaptive skills and quality of life, motor/neurologic functioning (neurologic assessment of choreiform movements, balance (Romberg’s test), diadochokinesis, finger-nose tapping, eye movements, muscle tone, reflexes, figure copying, and cognitive functioning. Vital signs during clinic visits (heart rate, systolic and diastolic blood pressure). Laboratory assessments including chemistry, hematology, and urinalysis.
- School-PANS (short version of PANS-scale rated by teacher or school assistant; Murphy, personal communication). Parental care load, e.g. sick leave, reduced working hours.
- The primary and secondary variables were assessed at Baseline, after 3 months, 5 months, 8 months, 12 months, and 18 months. In this report we present the 3-month primary outcomes and selected secondary outcomes.

### Procedures

After screening for inclusion in the trial, assessments were performed at Baseline (= 0 months), Visit 1 (= 1 month), Visit 2 (= 2 months), and Visit 3 (= 3 months). The IVIG infusions were given at Baseline, 1 and 2 months. All patients were assessed by two of the clinicians PH, EF, CG, MJ, the study nurses KJ and IV and the psychologist MO at clinical visits. The clinicians rated the PANS scale based on parent and patient interview, the CGI-S and CGI-I based on all available information, and school attendance based on parent and patient report. The psychologist rated the CY-BOCS.

Safety and tolerability were assessed by parent and patient report of clinical signs and symptoms and by vital signs (weight, blood pressure, pulse) at all visits, and also by complete blood counts including leucocyte differential, and alanine aminotransferase (ALAT) at baseline and Visit 3.

### Statistical analysis

Since this is an open-label exploratory uncontrolled trial, the sample size was not based on a statistical power calculation. The primary and secondary efficacy analyses and the safety analyses were performed on all included subjects (Intention-to-Treat population). There were no dropouts from the study. Changes from baseline to post-baseline visits were assessed using a two-tailed non-parametric comparison at a 0.05 significance level. Effect sizes are expressed as Cohen’s D with Hedge’s correction due to small sample size. Responder analysis was performed. Clinical response was defined as > 30% reduction in symptoms and impairment, respectively, as measured on the PANS scale, and as post-treatment values of 1 – 2 on the CGI-S and CGI-I scales. The distribution of continuous and interval scaled variables are presented as mean, standard deviation (SD), median, minimum, and maximum, and distribution of categorical variables in numbers, ratios, and percentages.

## Results

Table 1 shows participant demographics. Median age at PANS onset was 7.1 years (range 5.0 – 9.4), and at study entry (Baseline) 10.3 years (range 6.3 – 16.1). Four of the ten children had preexisting diagnosed neurodevelopmental disorders (Attention Deficit Hyperactivity Disorder (ADHD), autism/Asperger syndrome, autistic-like condition, unspecified epilepsy) and two had preexisting symptoms within these neurodevelopmental areas but did not meet full diagnostic criteria. A family history of autoimmune disorders was common, in first-degree relatives for one child and second-degree relatives for seven children (multiple sclerosis, rheumatoid arthritis, ulcerative colitis, coeliac disease, hypothyroidism, Guillain Barré syndrome, diabetes type 1, ankylosing spondylitis and autoimmune kidney disease). One patient had a verified streptococcus tonsillitis about two months before PANS symptom onset and one patient had positive rapid test for group A streptococcus after symptom onset. Three patients had clinically suspected streptococcal infection that coincided with symptom onset. Of these, one had impetigo, one had perianal streptococcal dermatitis and one developed fever whilst a sibling had diagnosed scarlet fever. Several children had concomitant medication at baseline, including SSRIs (*n* = 6) melatonin (*n* = 5), antipsychotics (*n* = 3; aripiprazole, clozapine), ADHD-medication (*n* = 3; guanfacine, methylphenidate) and antibiotics (*n* = 3; amoxicillin/clavulanic acid, phenoxymethylpenicillin). Additionally, two children were on NSAIDs and one on antihistamines at baseline. One patient had thyroid hormone treatment due to hypothyroidism. Two patients had received IVIG previously, more than one year ago.

Table 2 describes the baseline symptom profiles for our PANS cohort. All children had an acute symptom onset within a few days. At Baseline, all children had moderate to severe obsessive–compulsive symptoms according to the PANS scale. See also CY-BOCS scores in Fig. 6. One patient reported only subclinical symptoms on the CY-BOCS. The most common type of symptom was intrusive obsessional thoughts on symmetry and a need for things to feel, look or sound just right (90%). Other common obsessive–compulsive symptoms were intrusive worries about harming him/herself or others and a need to tell or confess (80%). Eating restriction was present in 80% of children.

As seen in Table 2 other common symptoms were anxiety, emotional lability and depression, irritability and aggressive behaviour, behavioural regression, difficulties with attention and learning, sensory symptoms, motor symptoms, sleep disturbance and dilated pupils. Children with sensory symptoms often had hypersensitivity towards sounds, light and clothing, and the most common motor symptoms were motor hyperactivity and simple tics. Hallucinations were only present in 2/10 children and no one showed signs of confusion.

Pain symptoms are not included in the PANS criteria but notably seven of the ten children had recurrent body pain when included in the study. Most children (6/10) had recurrent headaches, but some instead had joint pain or recurrent pain in their extremities or backs.

### Outcomes

All patients completed the first 3-month phase of the trial reported here. Significant changes with large effect sizes were obtained on all primary and secondary outcome measures when comparing Baseline to Visit 3 data.

### Primary outcomes (Table 3, Figs. 1, 2, 3, 4 and 5)

**Table 3:** Primary outcomes: PANS scale and CGI scores at Baseline, Visit 1 and Visit 3 (mean, SD). Secondary outcomes: CY-BOCS scores and school attendance at Baseline and Visit 3

PANS Scale (Total)	Mean	SD	ES	*P*-value
**Baseline**	81.2	7.82		
**Visit 1**	54.0	16.80		
**Visit 3**	47.4	14.64	2.64	= .005
*(n* = *10)*				
**PANS Scale (Symptom)**				
**Baseline**	41.3	5.29		
**Visit 1**	27.0	9.55		
**Visit 3**	24.9	7.00	3.15	= .005
* (n* = *10)*				
**PANS Scale (Impairment)**				
**Baseline**	39.8	3.39		
**Visit 1**	27.0	7.53		
**Visit 3**	22.5	7.91	2.07	= .005
* (n* = *10)*				
**CGI-S**				
**Baseline**	5.8	0.42		
**Visit 1**	3.9	0.74		
**Visit 3**	3.0	1.05	2.79	= .004
* (n* = *10)*				
**CGI-I**				
**Visit 1**	2.1	0.57		
**Visit 3**	1.8	0.79		
* (n* = *10)*				
**CY-BOCS (Total)**				
**Baseline**	24	7.80		
**Visit 3**	17.8	7.67	1.48	= .005
* (n* = *10)*				
**Days absent from school**				
**Baseline**	9.5	6.70		= .005
**Visit 1**	6.2	6.53		
**Visit 3**	2.7	3.53	1.06	
(*n* = 10)				

Means, SDs, Effect Size (ES, Cohen’s D with Hedges’ correction), *p*-values from non-parametric Wilcoxon signed ranks test

**Fig. 1:**
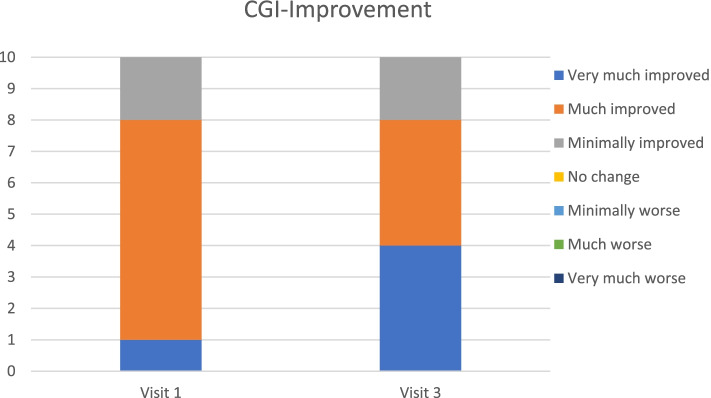
CGI-Improvement scores at Visit 1 and Visit 3

**Fig. 2:**
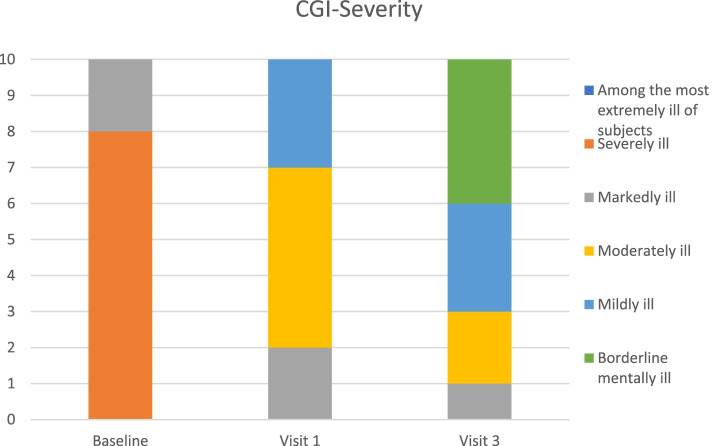
CGI-Severity scores at Baseline, Visit 1 and Visit 3

**Fig. 3:**
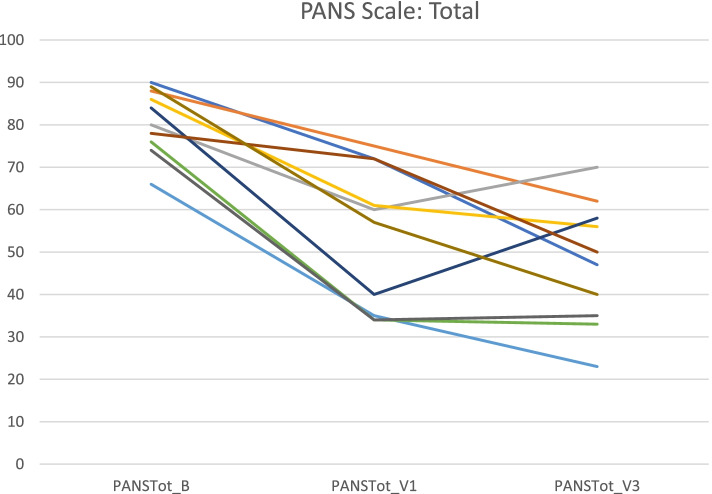
Total PANS Scale scores at Baseline, Visit 1 and Visit 3 for individual patients

**Fig. 4:**
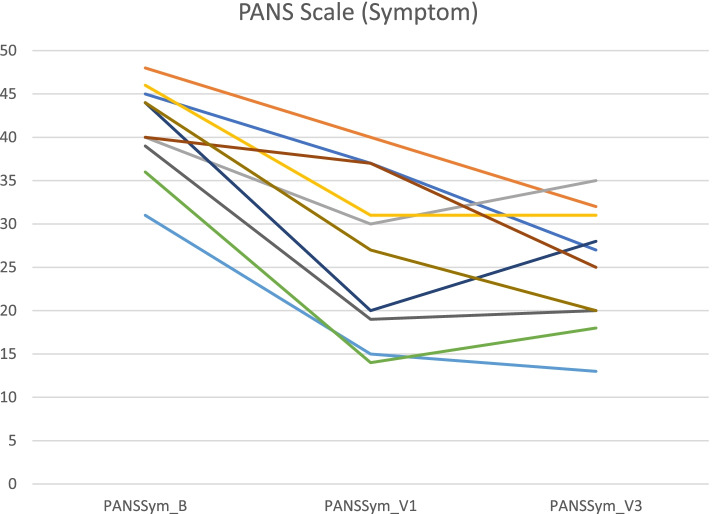
PANS Scale Symptom scores at Baseline, Visit 1 and Visit 3 for individual patients

**Fig. 5:**
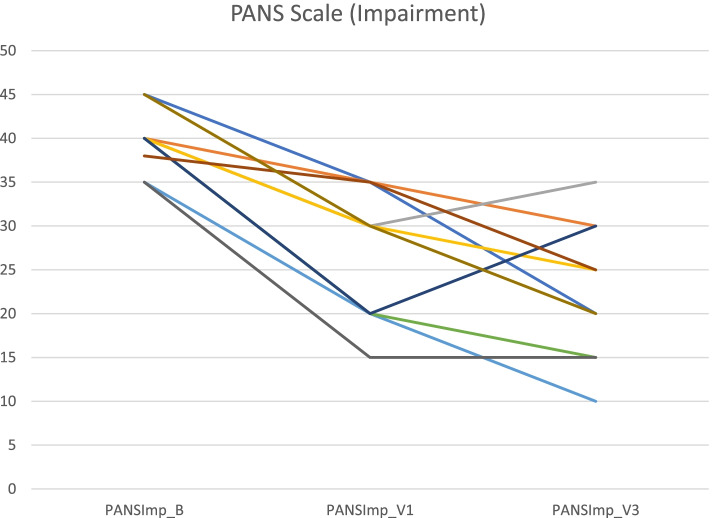
PANS Scale Impairment scores at Baseline, Visit 1 and Visit 3 for individual patients

Mean baseline PANS scale scores indicated severe illness (81.2/100). Mean scores improved to 54.0 (-33%) at Visit 1 (one month after the first IVIG treatment) and to 47.4 (-42%, ES 2·64, *p* = 0.005) at Visit 3 (one month after the third IVIG treatment). Nine patients were clinical responders at Visit 3 with more than 30% improvement on PANS scale total score. The improvements were global, i.e. there was no specific symptom in the PANS symptom complex that improved much more than the others. Baseline CGI-S scores indicated severe illness in most patients (mean 5·8). Mean CGI-S scores were 3.0 at Visit 3, corresponding to mild illness, and mean CGI-I scores were 1.8. Eight patients were responders measured by CGI-I scores 1 – 2 (much or very much improved). The most strict response definition was CGI-S scores 1 – 2 (borderline to not at all ill), which can be considered as remission of illness. By this measure, four patients were responders (remitted), and an additional three reached a CGI-S score of 3 (mildly ill) at Visit 3 (Figs. 1, 2, 3, 4 and 5). One patient showed good response at Visit 1 but increased symptoms at Visit 3 associated with an upper respiratory viral infection (total PANS Scale scores at Baseline, Visit 1 and Visit 3 were 84–40-58). One patient had good response for only 2–3 weeks after each IVIG followed by deterioration and therefore insufficient response at Visit 1 and 3 (PANS Scale scores 80–60-70).

### Secondary outcomes

#### CY-BOCS (Table 3 Fig. 6)

**Fig. 6:**
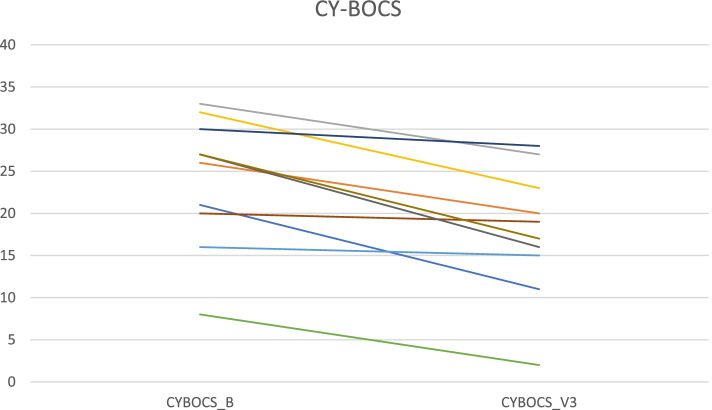
CY-BOCS scores at Baseline and Visit 3 for individual patients

Mean total scores improved from 24 at baseline to 17.8 at Visit 3 (-26%, ES 1.48, *p* = 0.005), indicating clinical response, as defined in previous research to be at least 25% total score reduction [20].

#### School attendance (Table 3)

At baseline the severe symptoms had considerable impact on the children’s school attendance but marked improvements in attendance were seen post-treatment. During 3 months before baseline the mean absence from school was 9.5 days per month (47% of full school time). After the first IVIG treatment it was 6.2 days per month (31%), and after the third IVIG treatment 2.7 days per month (13%). A comparison between baseline and visit 3 data indicated an improvement with a large effect size (ES 1.06, *p* = 0.005).

#### Adverse events

IVIG treatment side effects were frequent, transient, and of previously well-known type. After the first IVIG treatment two children had severe side effects (headache/neck pain/nausea/vomiting) one day post-treatment, followed by mild symptoms for 2 – 3 days. Even though some of these symptoms were temporarily severe, they were all expected, and none of the side effects were therefore categorized as Serious Adverse Events. Five children had moderate headache, neck or back pain, nausea, or irritability lasting 1 – 5 days, and the remaining three children had mild headache for hours to a few days. Side effects were less severe or non-existent after the second treatment (moderate headache, nausea, or stomach pain (*n* = 3), mild headache and/or nausea (*n* = 6), duration 1–5 days) and after the third treatment (moderate headache and nausea (*n* = 2), mild headache, nausea, fatigue (*n* = 6), duration a few hours to 7 days). No clinically important changes in vital signs (weight, blood pressure, pulse) or safety laboratory parameters were observed, with the exception that one child developed mild temporary anemia after the second IVIG. One child had a brief allergic reaction during the third IVIG (treated with steroids).

## Discussion

This open-label trial of three monthly 2 g/kg IVIG treatments in ten children with PANS is the first IVIG study to report broad baseline and follow-up data on global severity and functioning and detailed symptom development according to the defined symptom criteria of PANS.

Considerable improvements of PANS global symptoms and impairment were seen on the PANS Scale, CGI-S, CGI-I, and improved OCD symptoms on the CY-BOCS scale, lasting at least one month after the third IVIG treatment (at Visit 3). All children had improved at Visit 1 (one month after the first IVIG treatment). At Visit 3 eight of the ten children had lasting improvements, but two showed symptom rebounds. One of these had good but transient responses lasting only 2–3 weeks after each IVIG, followed by deteriorations, and the other had a concurrent viral infection at Visit 3. At baseline the severe symptoms had considerable impact on the children’s school attendance but marked improvements in attendance were seen post-treatment. School absence during 3 months before baseline was 47% of the school days/month, compared to 13% after the third IVIG treatment.

The main side effects were of a previously well-known type, i.e. transient headache, neck pain, nausea/vomiting (mild to severe), stomach pain, mild transient anemia, and brief allergic reaction. None of the adverse events led to treatment discontinuation. All patients completed the whole trial period.

Psychopharmacological treatment was common in our cohort of children. At baseline 60% had SSRI-medication and 50% had medication for insomnia. Antipsychotics and ADHD medications were used by 30% respectively. These are the standard treatments used in children presenting with OCD, tics, hyperactivity, sleeping and behavioural problems. Nevertheless, participants had high baseline scores on PANS scale (81.2/100) and CGI-S (5.8/7) indicating severe illness and a limited effect of previously prescribed drugs. The considerable improvements seen after IVIG treatment in both symptoms and functioning during the trial point to a beneficial role of IVIG in controlling PANS symptoms and suggests that PANS symptoms may be immune-mediated.

The hypothesized neurological background of PANS/PANDAS is an immune-mediated brain inflammatory disorder involving basal ganglia structures [10, 21], but previous treatment trials with immunoglobulin have shown conflicting results. A placebo-controlled single-dose IVIG treatment trial of infection-triggered exacerbations of neuropsychiatric symptoms in children with OCD and tic disorders was performed in 1999 [13]. This study, comparing three interventions, i.e. IVIG, plasma exchange and placebo, showed that IVIG and plasma exchange were superior to placebo. A recent double-blind placebo-controlled study [14] with a single dose of IVIG or placebo in children with PANDAS showed some response in both the IVIG and the placebo group during the double-blind phase, but no significant difference between groups. This highlights the difficulty in distinguishing between effects of placebo and active treatment. Interestingly, in a following phase of that study non-responders received an open-label IVIG treatment, after which robust mean improvements in OCD symptoms (CY-BOCS) and CGI-I were observed.

The obvious benefits in general of randomized controlled trials (RCTs) may have to be reconsidered in certain invasive interventions and study populations. For example, the problems to use placebo in long-lasting intravenous treatments would require certain ethical considerations. Therefore placebo-controlled double-blind RCTs which minimize risk and time on placebo through cross-over design would have a clear advantage.

In addition to the ethical problem of treating children with an inactive, intravenously administered placebo, it is also problematic to randomize children with complex neurodevelopmental/neuropsychiatric disorders with different symptom combinations and severities.

Although RCTs provide the best study design in many types of intervention studies, several authors have pointed out that this design may have serious limitations when studying complex multifactorial determined developmental disorders [22–24]. For example, a Cochrane report on the early intensive behavioral intervention for young children with autism spectrum disorders also included non-randomized trials, showing that designs other than RCT can be of importance [25].

Consequently, there are some open-label studies published on IVIG treatment in children meeting criteria for PANDAS or PANS [15, 16]. Melamed et al. 2021, in their open-label study of 21 children with PANS discussed the limitations of open trials and commented that if PANS was not an autoimmune, autoinflammatory disease, then an immunomodulatory intervention, such as IVIG, should not have any impact on psychometric and clinical measurements.

Our study design accorded with the seven items outlined in the first version of the Methodological Index for Non-Randomized Studies (MINORS), developed by Slim et al. [26]. This method may be used when there are specific methodological difficulties in conducting randomized trials and when observational or non-randomized studies must be used. The seven items are: 1. A stated aim of the study, 2. Inclusion of consecutive patients, 3. Prospective collection of data, 4. Endpoint appropriate to the study aim, 5. Unbiased evaluation of endpoints, 6. Follow-up period appropriate to the major endpoint and 7. Loss to follow up not exceeding 5%.

Open-label trials cannot, however, with certainty distinguish pure treatments effects from effects of time, other factors, or expectancy bias. To strengthen the evidence in our study, we therefore included several outcomes and ratings from different sources, focusing on outcomes that reflect a real-life impact of treatment. In addition to results from the PANS scale, CGI-S and CGI-I, CY-BOCS and measures of school attendance published here, long-term data on functioning and quality of life will be published later.

All 10 children in our study group had an abrupt onset of PANS symptoms and were severely ill. Heredity for autoimmune disease was reported for 7 children. Four and two children, respectively, had preceding diagnoses of neurodevelopmental disorders of varying severities or obvious symptoms within these areas without meeting criteria for such diagnoses. Also, regarding other features, they all had their specific characteristics, making a randomization process hazardous.

## Limitations

The main limitations of this trial include a small sample size, the non-randomized uncontrolled design, the lack of a control group, and a heterogeneous patient background in terms of preexisting disorders, medication and duration of illness. The main strengths of the study are the evaluation with broad in-depth outcomes of high real-life validity, ratings made both by investigators, parents and children, and the data collected on school attendance. The results of the long-term 18-month follow-up included in the trial will be reported in a forthcoming publication.

## Conclusions

This open-label prospective IVIG treatment trial in 10 children with PANS demonstrated substantial improvements in PANS symptom severity and impairment (including OCD symptoms), global functioning and school attendance after 3 monthly IVIG treatments. From severe illness at baseline, 9 patients were clinical responders with > 30% improvement, and 7 patients improved to mild illness or remission. Side effects of previously well-known type (mostly headache, nausea) were temporarily moderate to severe, but no patient discontinued the trial due to side effects.

## Data Availability

The datasets used and/or analysed during the current study are available from the corresponding author on reasonable request.

## Abbreviations

ADHD: Attention Deficit Hyperactivity Disorder
AEs: Adverse Events
ALAT: Alanine aminotransferase
ASAT: Aspartate aminotransferase
CGI-S: Clinical Global Impression – Severity scale
CGI-I: Clinical Global Impression – Improvement scale
CY-BOCS: Children’s Yale-Brown Obsessive Compulsive Scale
GNC: Gillberg Neuropsychiatry Centre
IVIG: Intravenous immunoglobulin
MINORS: Methodological Index for Non-Randomized Studies
NSAIDs: Nonsteroid Anti-Inflammatory Drugs
OCD: Obsessive-Compulsive Disorder
OPHELIA: On PANS: Holistic Epidemiology, Longitudinal Immunotherapies, Additional treatments
PANS: Paediatric Acute-onset Neuropsychiatric Syndrome
PANDAS: Paediatric Autoimmune Neuropsychiatric Disorders Associated with Streptococcal infections
RCT: Randomized Controlled Trial
SAEs: Serious Adverse Events
SSRI: Selective Serotonin Reuptake Inhibitors

## References

[1] Swedo SE, Leckman JF, Rose NR (2012). From research subgroup to clinical syndrome: modifying the PANDAS criteria to describe PANS (Pediatric Acute-onset Neuropsychiatric Syndrome). Pediatr Therapeut.

[2] Frankovich J, Thienemann M, Pearlstein J (2015). Multidisciplinary clinic dedicated to treating youth with pediatric acute-onset neuropsychiatric syndrome: presenting characteristics of the first 47 consecutive patients. J Child Adolesc Psychopharmacol.

[3] Chang K, Frankovich J, Cooperstock M (2015). Clinical evaluation of youth with pediatric acute-onset neuropsychiatric syndrome (PANS): recommendations from the 2013 PANS Consensus Conference. J Child Adolesc Psychopharmacol.

[4] Johnson M, Fernell E, Preda I (2019). Paediatric acute-onset neuropsychiatric syndrome in children and adolescents: an observational cohort study. Lancet Child Adolesc Health.

[5] Gromark C, Harris RA, Wickström R (2019). Establishing a pediatric acute-onset neuropsychiatric syndrome clinic: baseline clinical features of the pediatric acute-onset neuropsychiatric syndrome cohort at Karolinska Institutet. J Child Adolesc Psychopharmacol.

[6] Swedo SE, Frankovich J, Murphy TK (2017). Overview of treatment of pediatric acute-onset neuropsychiatric syndrome. J Child Adolesc Psychopharmacol.

[7] Mataix-Cols D, Frans E, Pérez-Vigil A (2018). A total-population multigenerational family clustering study of autoimmune diseases in obsessive-compulsive disorder and Tourette’s/chronic tic disorders. Mol Psychiatry.

[8] Thienemann M, Murphy T, Leckman J (2017). Clinical management of pediatric acute-onset neuropsychiatric syndrome: part I—psychiatric and behavioral interventions. J Child Adolesc Psychopharmacol.

[9] Chiarello F, Spitoni S, Hollander E, MatucciCerinic M, Pallanti S (2017). An expert opinion on PANDAS/PANS: highlights and controversies. Int J Psychiatry Clin Pract.

[10] Frankovich J, Swedo S, Murphy T (2017). Clinical management of pediatric acute-onset neuropsychiatric syndrome (PANS): Part II—Use of immunomodulatory therapies. J Child Adolesc Psychopharmacol.

[11] Sigra S, Hesselmark E, Bejerot S (2018). Treatment of PANDAS and PANS: a systematic review. Neurosci Biobehav Rev.

[12] Johnson M, Ehlers S, Fernell E (2021). Anti-inflammatory, antibacterial and immunomodulatory treatment in children with symptoms corresponding to the research condition PANS (Pediatric Acute-onset Neuropsychiatric Syndrome): A systematic review. PLoS One.

[13] Perlmutter SJ, Leitman SF, Garvey MA (1999). Therapeutic plasma exchange and intravenous immunoglobulin for obsessive-compulsive disorder and tic disorders in childhood. Lancet.

[14] Williams KA, Swedo SE, Farmer CA (2016). Randomized, controlled trial of intravenous immunoglobulin for pediatric autoimmune neuropsychiatric disorders associated with streptococcal infections. J Am Acad Child Adolesc Psychiatry.

[15] Melamed I, Kobayashi RH, O’Connor M (2021). Evaluation of intravenous immunoglobulin in pediatric acute-onset neuropsychiatric syndrome. J Child Adolesc Psychopharmacol.

[16] Pavone P, Falsaperla R, Cacciaguerra G (2020). PANS/PANDAS: clinical experience in IVIG Treatment and state of the art in rehabilitation approaches. NeuroSci.

[17] Pediatric acute neuropsychiatric symptoms (PANS) scale, parent version. (https://pandasnetwork.org/wp-content/uploads/2018/11/pandas_pans_scale.pdf). Accessed 10 Jan 2022.

[18] Guy W (1976). CDEU Assessment manual for psychopharmacology. Revised.

[19] Scahill L, Riddle MA, McSwiggin-Hardin M (1997). Children’s Yale-Brown ObsessiveCompulsive Scale: reliability and validity. J Am Acad Child Adolesc Psychiatry.

[20] Storch EA, Lewin AB, De Nadai AS (2010). Defining treatment response and remission in obsessive-compulsive disorder: a signal detection analysis of the Children’s Yale-Brown Obsessive-Compulsive Scale. J Am Acad Child Adolesc Psychiatry.

[21] Hornig M (2013). The role of microbes and autoimmunity in the pathogenesis of neuropsychiatric illness. Curr Opin Rheumatol.

[22] Graham HK (2007). The trials of trials. Dev Med Child Neurol.

[23] Rosenbaum P (2010). The randomized controlled trial: An excellent design, but can it address the big questions in neurodisability?. Dev Med Child Neurol.

[24] Mesibov GB, Shea V (2011). Evidence-based practices and autism. Autism..

[25] Reichow B, Barton EE, Boyd BA (2012). Early intensive behavioral intervention (EIBI) for young children with autism spectrum disorders (ASD). Cochrane Database Syst Rev.

[26] Slim K, Nini E, Forestier D (2003). Methodological Index for Non-Randomized Studies (MINORS): development and validation of a new instrument. ANZ J Surg.

